# Supplementary material for: Coordinated regulation of pH alkalinization by two transcription factors promotes fungal commensalism and pathogenicity
Source: Nat Commun. 2025 Aug 22;16:7855. doi: 10.1038/s41467-025-62953-x (PMC12373999; doi:10.1038/s41467-025-62953-x)
Supplement: Supplementary file 1 — Supplementary information [file 41467_2025_62953_MOESM1_ESM.pdf]

Supplementary information

Coordinated regulation of pH alkalinization by two transcription factors promotes fungal commensalism and pathogenicity

Xinhua Huang[1*,#], Guangsheng Chen[2,#], Lei Wu[1,6,#], Yun Zou[1,6#], Luyao Zhang[1,6], Shanshan Li[1], Kunlin Li[1,6], Zaijie Jiang[1], Yuping Zhang[1], Xiaoqing Chen[1,6], Winnie Shum[3], Jianbiao Dai[3], Huichang Huang[1], Munika Moses[1,6], Xianwei Wu[1,6], Yuanyuan Wang[1], Tong Jiang[1,6], Zhiyi He[2], Qing Guo[4], Wenwen Xue[5], Hao Li[7*], Changbin Chen[1]*

[1] Joint Laboratory for Biomedical Research and Pharmaceutical Innovation, The Unit of Pathogenic Fungal Infection & Host Immunity, Shanghai Institute of Immunity and Infection, Chinese Academy of Sciences, Shanghai 200031, China.

[2] Department of Respiratory and Critical Care Medicine, The First Affiliated Hospital of Guangxi Medical University, Nanning, Guangxi, China.

[3] USJ-Kong Hon Academy for Cellular Nutrition and Health, University of Saint Joseph, Macau, China.

[4] Reproductive Medicine Center, The Fourth Hospital of Shijiazhuang (Affiliated Obstetrics and Gynecology Hospital of Hebei Medical University), Shijiazhuang, Hebei, China.

[5] Nanjing Advanced Academy of Life and Health, Nanjing, China.

[6] University of Chinese Academy of Sciences, Beijing, China.

[7] Organ Transplantation Clinical Medical Center of Xiamen University, Department of General Surgery, Xiang'an Hospital of Xiamen University, School of Medicine, Xiamen University, Xiamen, China

Mutant ID	Gene Name / Systematic Name	Gene Description
M39	*ALI1*	Putative NADH-ubiquinone oxidoreductase
M58	*MCI4*	Putative NADH-ubiquinone dehydrogenase
M60	*PEX8*	Putative peroxisomal biogenesis factor
M162	*orf19.9078*	Has domain(s) with predicted aminoacyl-tRNA hydrolase activity
M176	*orf19.10861*	Protein similar to a mitochondrial complex I intermediate-associated protein
M185	*RBD1*	Rhomboid-like protein
M338	*ZCF5*	Zn(II)2Cys6 transcription factor
M361	*YEA4*	Putative uridine diphosphate-N-acetylglucosamine (UDP-GlcNAc) transporter
M508	*PTC2*	Protein phosphatase of the Type 2C-related family (serine/threonine-specific)
M542	*IFF5*	Putative GPI-anchored protein
M584	*CCN1*	G1 cyclin
M628	*PST2*	Flavodoxin-like protein involved in oxidative stress protection and virulence
M634	*FAT1*	Predicted enzyme of sphingolipid biosynthesis
M690	*GAT2*	Transcription factor
M674	*GRR1*	F-box protein component of the SCF ubiquitin-ligase complex required for cell cycle progression
M851	*SIT4*	Serine/threonine protein phosphatase catalytic subunit
M944	*SIN3*	Transcriptional corepressor involved in histone deacetylase recruitment
TF016	*BAS1*	Putative Myb-like transcription factor
TF106	*CSR1*	Transcription factor
TF110	*ROB1*	Zn(II)2Cys6 transcription factor
TF127	*ISW2*	An ATPase involved in chromatin remodeling;
TF132	*AHR1*	Zn(II)2Cys6 transcription factor
TF155	*DAL81*	Zn(II)2Cys6 transcription factor

Supplementary Fig. 1 Candidate *C. albicans* mutants with alkalinization defects. Shown are mutants displaying reduced *in vitro* alkalinization of phenol red-containing medium 199 (initial pH 4.0), identified from a screen of 674 mutant strains.

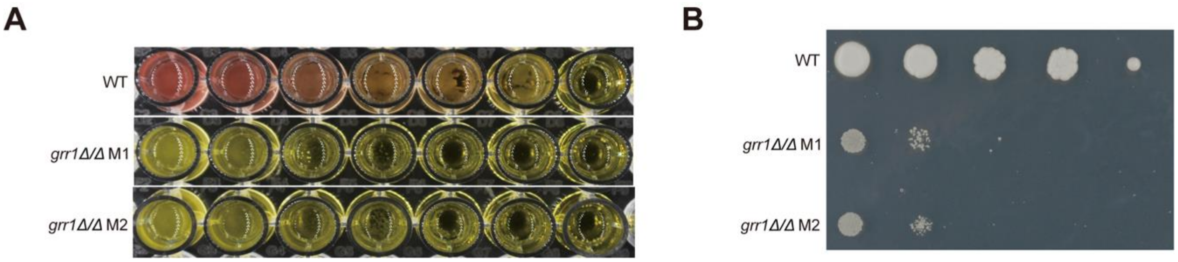

Supplementary Fig. 2 Alkalinization and growth phenotypes of *grr1Δ/Δ* mutant. (A) The alkalinization assay was performed following the procedures described in Fig. 1B. **(B)** WT and two independent *grr1Δ/Δ* mutant strains were grown overnight in YPD, then adjusted to an OD_{600} of 1.0. These cultures were serially diluted at a ratio of 1:10, and a 2 µl of each dilution was spotted onto YNB +1% CAA agar plates. Plates were incubated at 30°C and photographed after 2 days of growth.

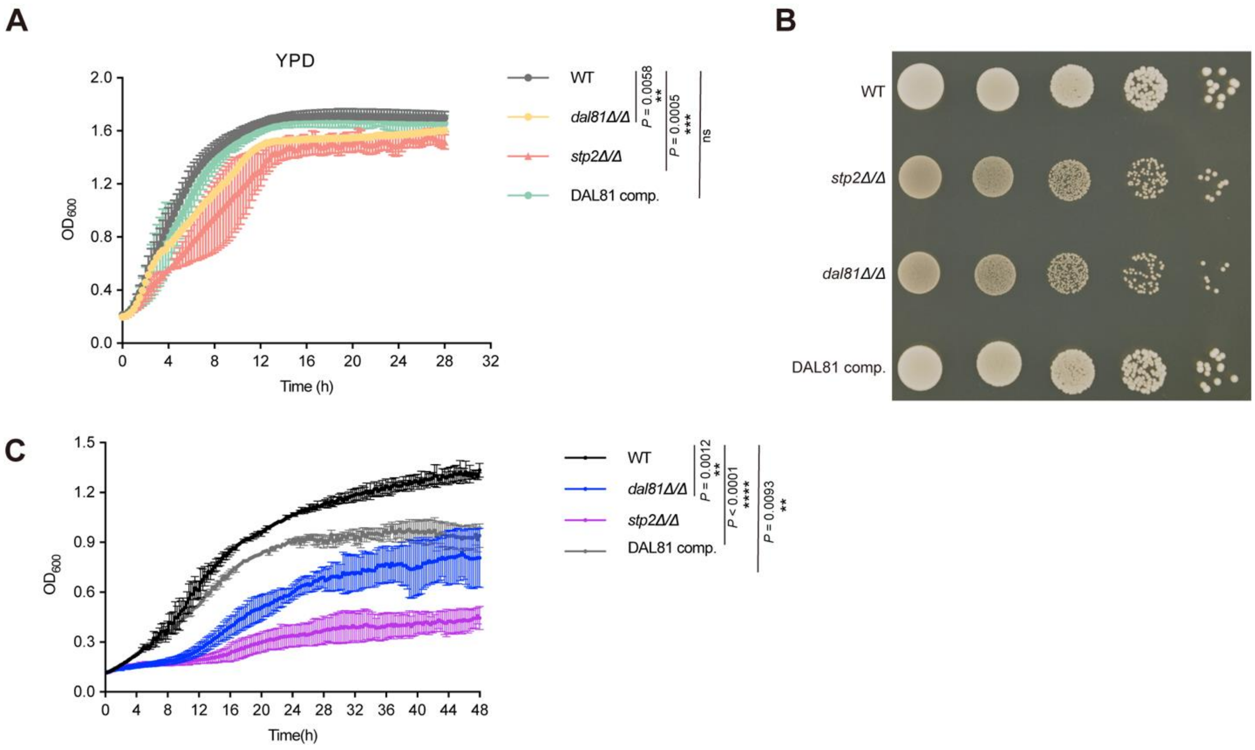

Supplementary Fig. 3 ***C. albicans* *dal81Δ/Δ* mutant cells exhibit mild growth defect in YPD but significantly severe impairments in YNB + 1 % CAA. (A)** *C. albicans* strains of indicated genotypes were grown in liquid YPD at 30°C. OD_{600} values were recorded every 15 min for 28 h using a BioTek Synergy 2 Multi-mode Microplate Reader. **(B)** Overnight YPD cultures were adjusted to OD_{600} =1.0, serially diluted, and 2 µl of each suspension was spotted onto YPD agar plates. Plates were incubated at 30°C and photographed after 3 days of growth. **(C)** Growth rates of mutants with significant growth reduction relative to wild-type in YNB + 1 % CAA (initial pH 4.0) at 30°C. OD_{600} values were measured every 15 min for 48 h. Data are mean ±SD from three biological replicates. Statistical significance was determined by one-way ANOVA with Turkey's post hoc test at 16h (A) and 48h (C). ns, not significant. Source data are provided in the Source Data file. Source data for (A, C) are provided in the Source Data file.

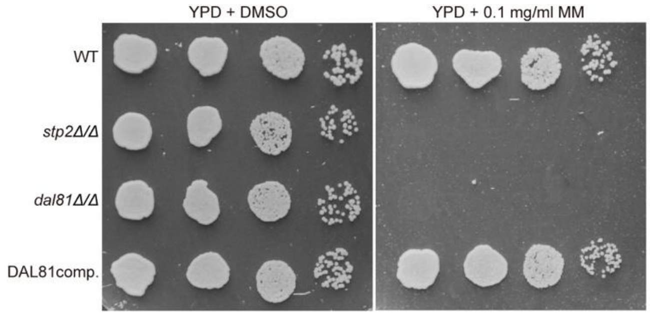

Supplementary Fig. 4 The *dal81Δ/Δ* mutant exhibits a diminished ability to take up amino acids. Growth phenotypes of strains with indicated genotypes were assessed by spotting serial dilutions of WT, *dal81Δ/Δ*, *stp2Δ/Δ*, DAL81 comp. strains onto SC medium and SC medium supplemented with metsulfuron methyl (MM). Plates were incubated at 30°C for 2 days and then photographed.

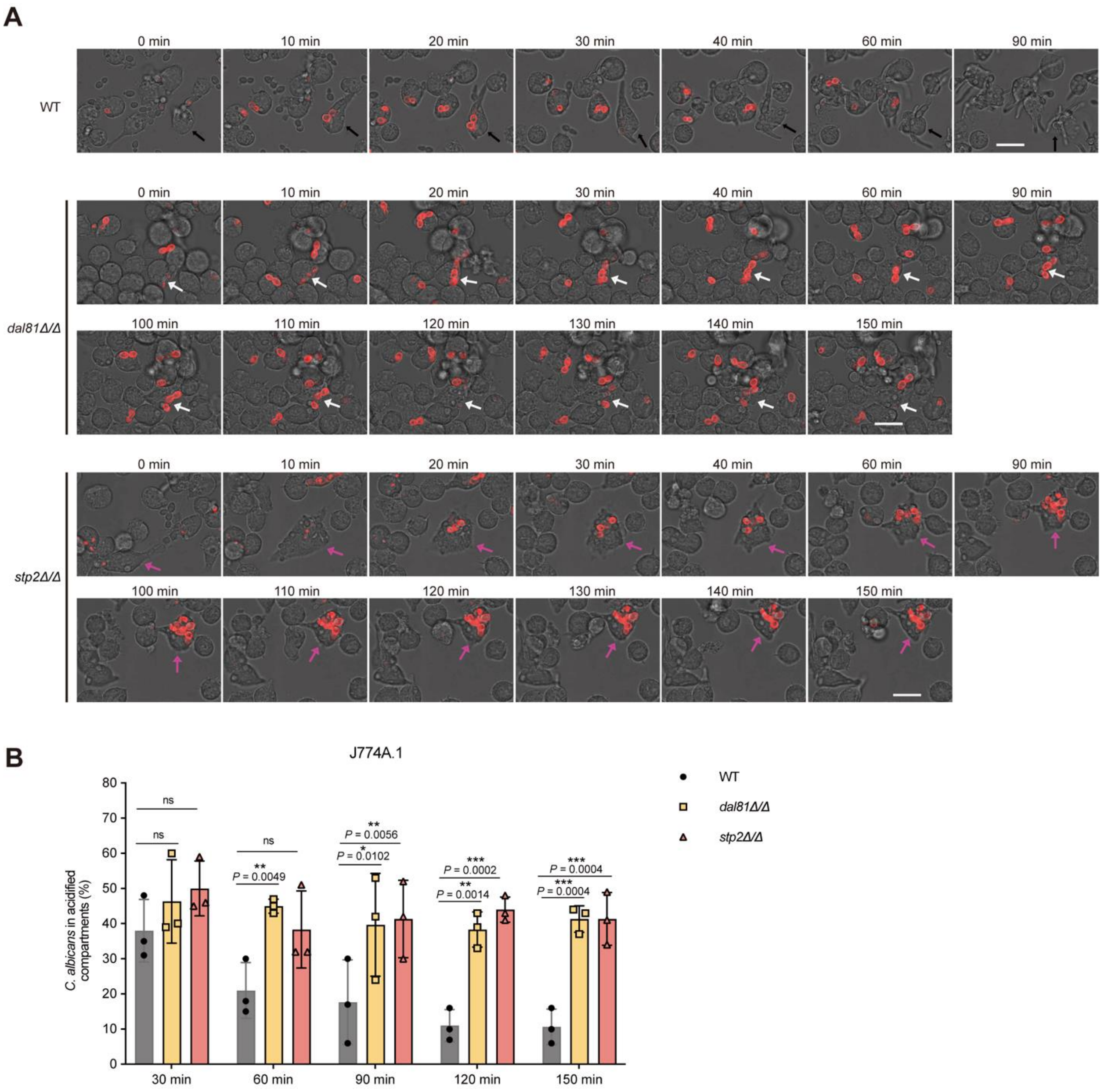

Supplementary Fig. 5 Temporal dynamics of phagosome acidification in J774A.1 macrophages infected with *C. albicans*. **(A)** J774A.1 macrophages were infected with log-phase wild-type, *dal81Δ/Δ* or *stp2Δ/Δ* strains. Interactions between fungal cells and macrophages were visualized via time-lapse microscopy. Representative images are shown for each group. Arrows indicate the progression of fungal cells within a single macrophage. Scale bar, 20 μm. **(B)** Quantification of acidified intracellular compartments in J774A.1 cells containing WT, *dal81Δ/Δ* or *stp2Δ/Δ* strains. At least 100 fungal cells were evaluated for pHrodo positive signals. Data are mean ±SD of three biological replicates. Statistical significance was determined using two-way ANOVA with Turkey's test. ns, not significant. Source data for (B) are provided in the Source Data file.

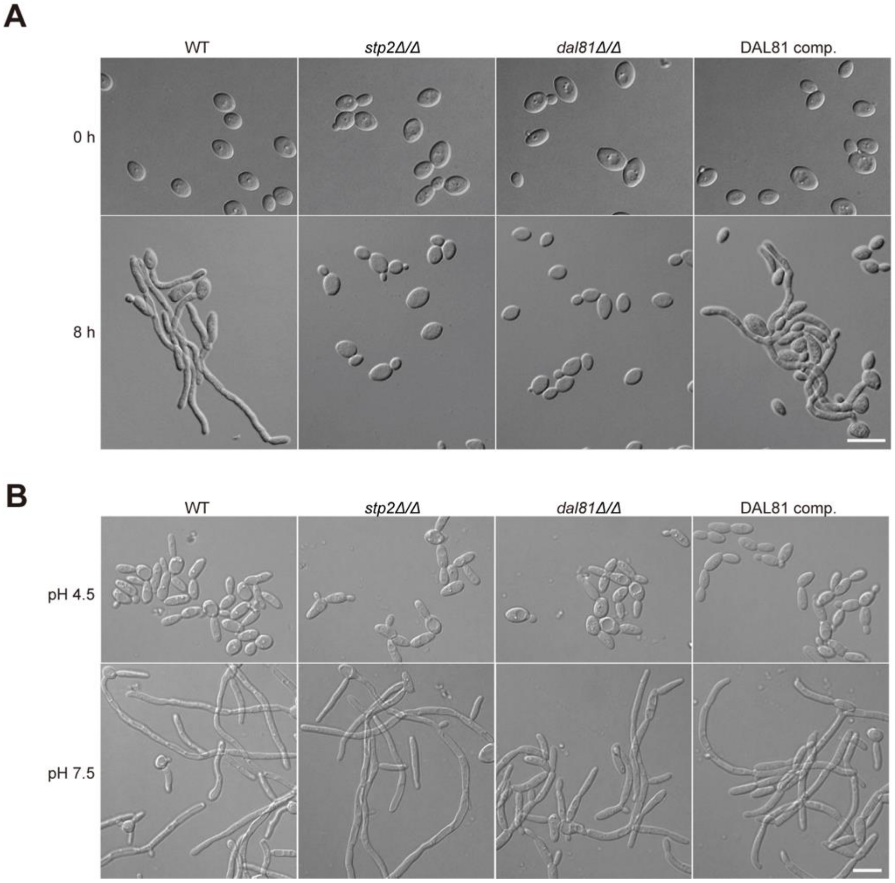

Supplementary Fig. 6 *DAL81*-deficient cells failed to switch to hyphae due to their incapability to alkalinize the environmental pH. (A) Strains of indicated genotypes were grown overnight in YPD, diluted to OD_{600} = 0.2 in YNB+1%CAA (initial pH 4.5). Cellular morphology was assessed via photomicrographs after 8 h. **(B)** Overnight YPD cultures were transferred to medium 199 buffered at pH 4.5 or 7.5, incubated at 37°C, and samples were collected and photographed after 5 h. Scale bar, 10 μm.

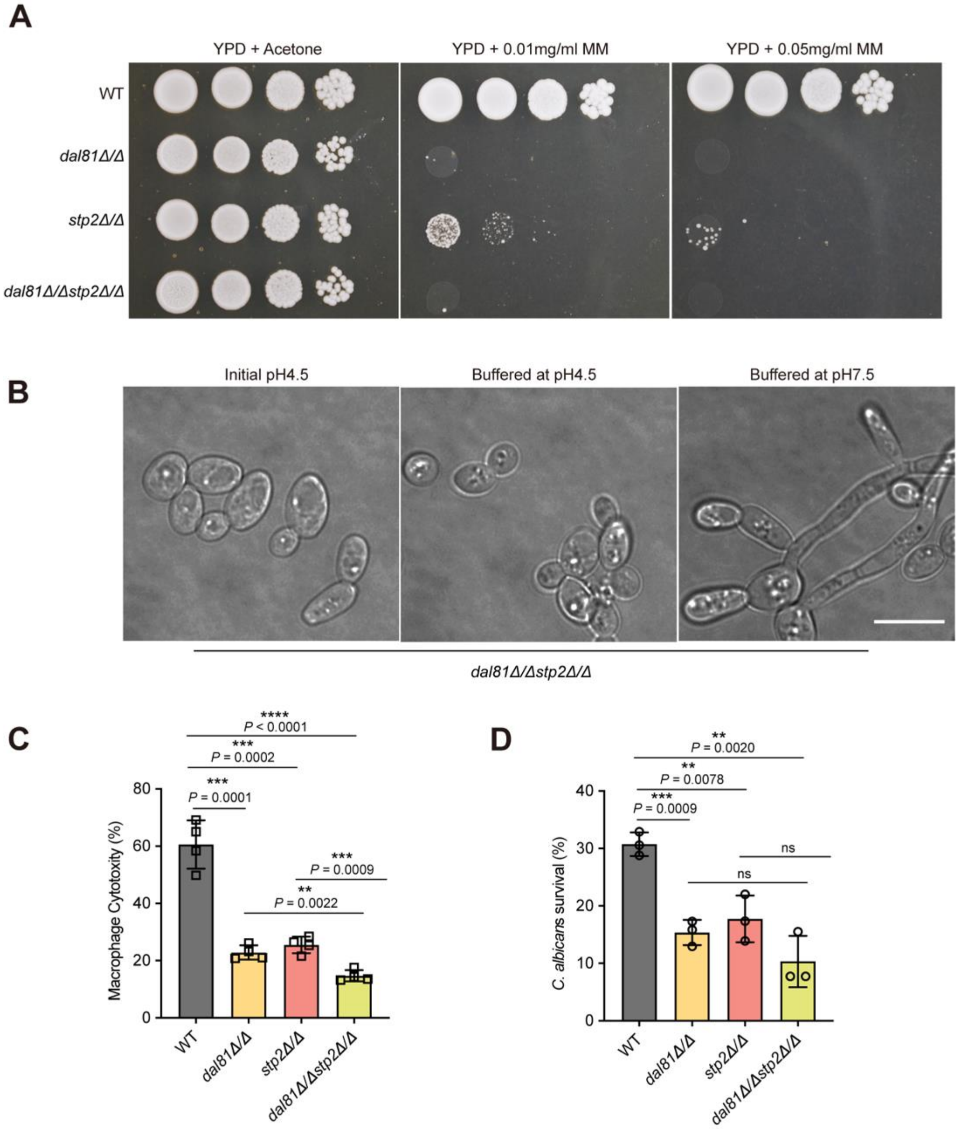

Supplementary Fig. 7 **Phenotypic assessment of the *dal81Δ/Δ stp2Δ/Δ* double mutant.** **(A)** Growth phenotypes of strains with indicated genotypes. Serial dilutions of WT, *dal81Δ/Δ*, *stp2Δ/Δ* and *dal81Δ/Δ stp2Δ/Δ* strains were spotted onto YPD medium and YPD medium containing MM. Plates were incubated at 30°C for 2 days and photographed. **(B)** Overnight YPD cultures were diluted to OD_{600} =0.2 in YNB + 1% CAA (initial pH 4.5) or medium 199 buffered at either pH 4.5 or 7.5. Cells were incubated at 37°C and samples were collected and photographed after 5 h or 8 h. Scale bar, 10 µm. **(C)** Macrophage cytotoxicity was determined by measuring LDH release after 5h incubation with fungal cells at MOI = **3**. **(D)** Endpoint dilution assay to assess strain sensitivity in macrophage killing. Data are mean ±SD of three biological replicates. Statistical significance was determined using an unpaired two-tailed Student's *t*-test (C, D). ns, not significant. Source data for (C, D) are provided in the Source Data file.

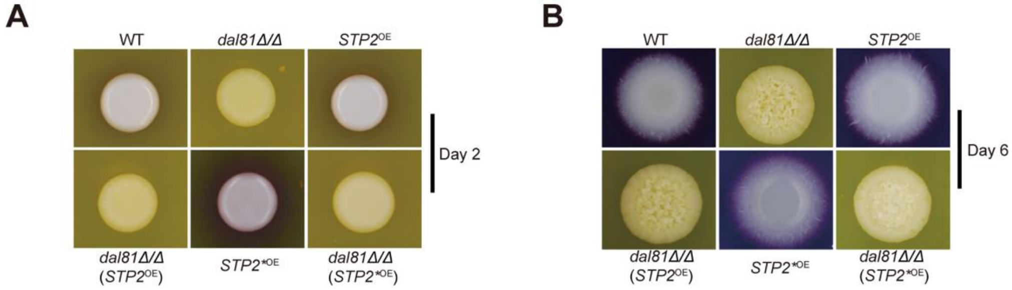

Supplementary Fig. 8 Overexpression of *STP2* in the *dal81Δ/Δ* mutant failed to rescue its alkalinization defects. The alkalinization assay was performed as described in Fig. 3E, with the exception that the plates were photographed after 2 days (A) or 6 days (B).

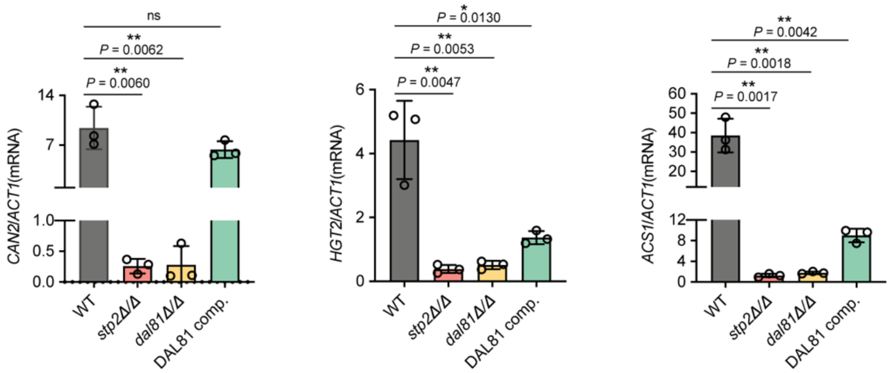

Supplementary Fig. 9 RT-qPCR analysis of three target genes regulated by Dal81 and Stp2. WT, *dal81Δ/Δ*, *stp2Δ/Δ,* DAL81 comp. strains were grown overnight in YPD, centrifuged, washed with water, and diluted to OD_{600}= 0.1 in 10 ml unbuffered medium 199 (initial pH 4.0). Cells were incubated at 37°C until the pH of WT cultures reached 5.0. Data are mean ±SD of three biological replicates. Statistical significance was determined using an unpaired two-tailed Student's *t*-test. ns, not significant. Source data are provided in the Source Data file.

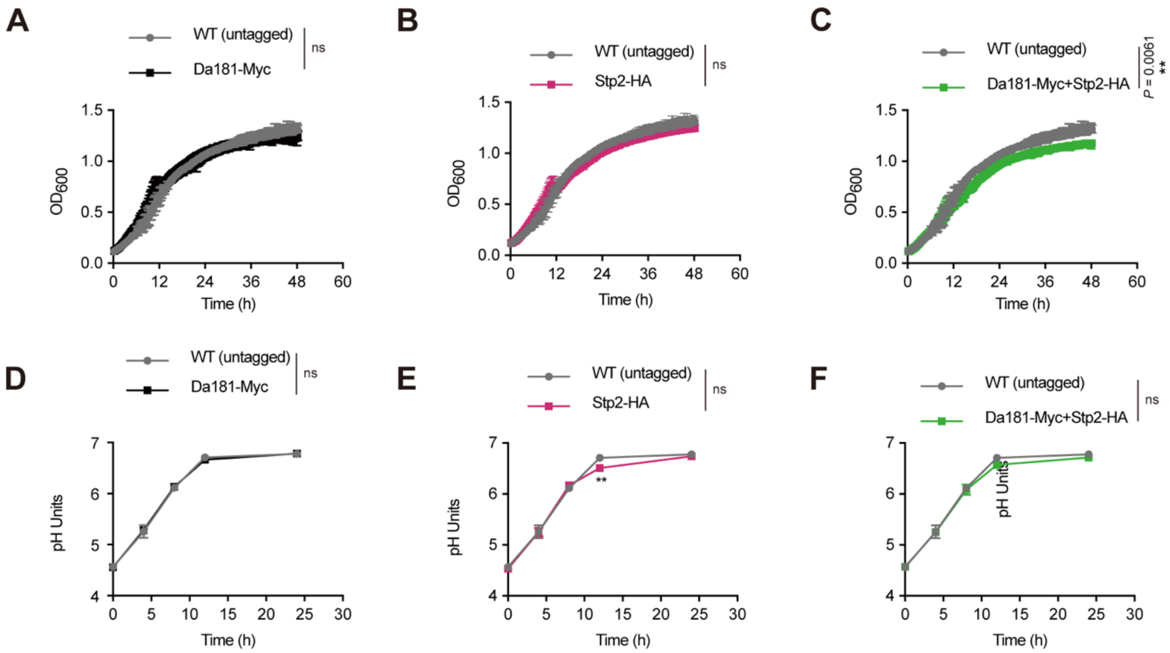

Supplementary Fig. 10 Growth and alkalinization phenotypes of tagged and untagged strains. (A-C) Strains with indicated genotypes were grown in YNB + 1 % CAA (initial pH 4.5) at 30°C. OD_{600} values were recorded every 15 min for 48 h using a BioTek Synergy 2 Multi-mode Microplate Reader. **(D-F)** Cells of indicated strains were grown in YNB+1% CAA medium (initial pH 4.5) at 37°C. Lines represent mean ±SD of three biological replicates. Statistical significance was determined using an unpaired two-tailed Student's *t*-test at 48h (A-C) and 24h (D-F). ns, not significant. Source data are provided in the Source Data file.

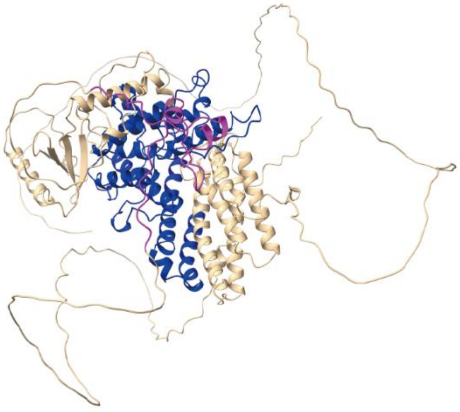

Supplementary Fig. 11 AlphaFold structural prediction of *C. albicans* Dal81. The predicted structure of *C. albicans* Dal81 is shown, with domains color-coded: the Zn_2/Cys_6 DNA-binding domain is highlighted in purple, and the fungal-specific transcription factor domain is shown in blue.

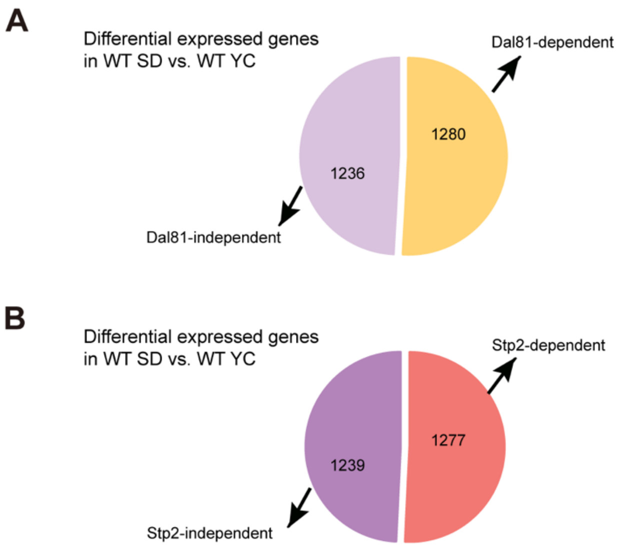

Supplementary Fig. 12 Schematic diagram illustrating DEGs after alkaline pH induction either in a Dal81-dependent or Stp2-dependent manner. pH alkalinization-responsive genes were identified based on the fold change in gene expression in SC5314 cells cultured in alkaline pH-inducing medium (YC) relative to SD medium. **(A)** Dal81-dependent genes and **(B)** Stp2-dependent genes were defined as those where the fold change in the *dal81Δ/Δ* or *stp2Δ/Δ* strain differed by ≥2-fold compared to the wild-type strain (SN250). Genes failing to meet this threshold were classified as Dal81-independent or Stp2-independent, respectively.

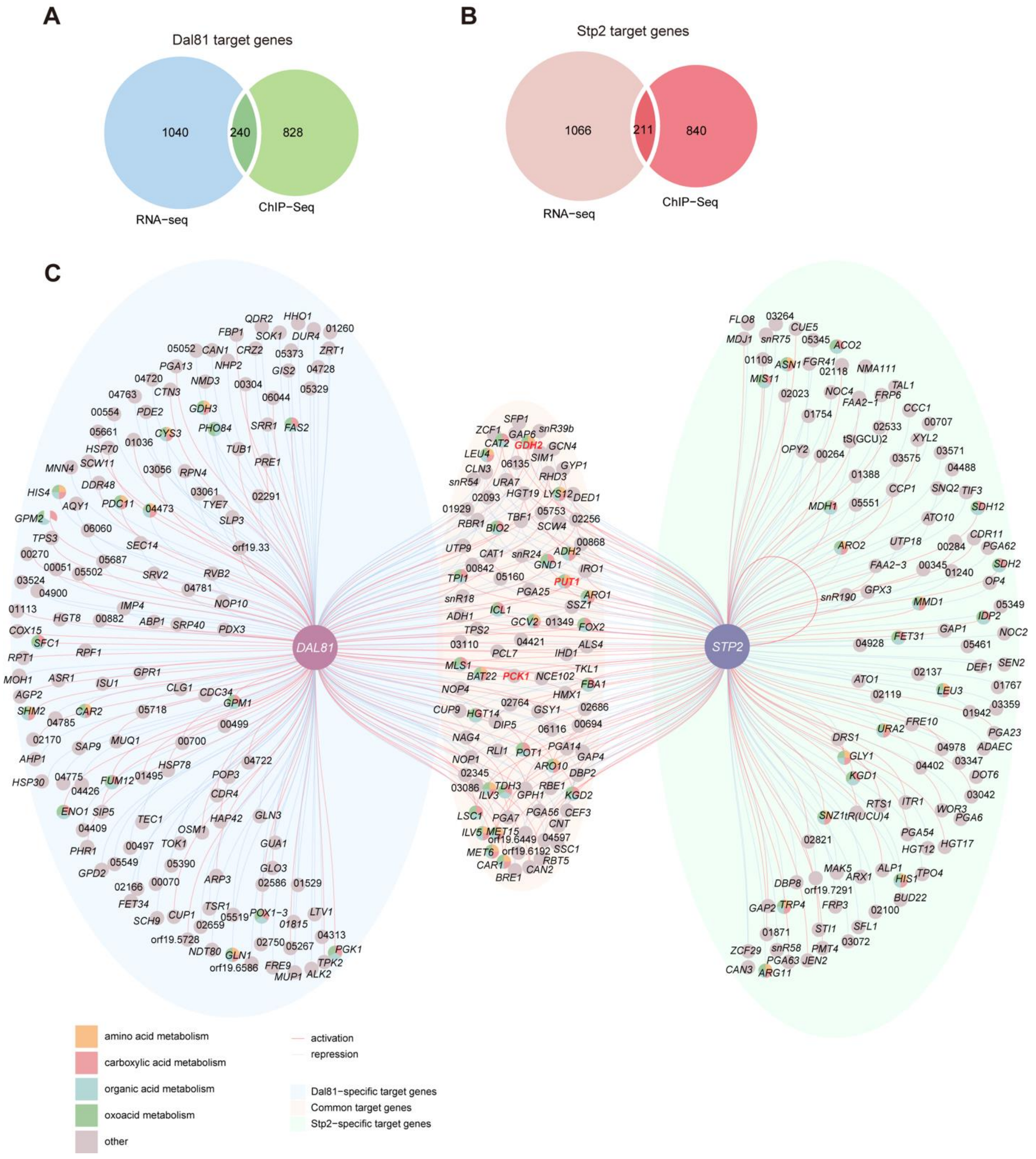

Supplementary Fig. 13 Direct gene regulation by Dal81 and Stp2. (A-B) Venn diagram showing overlaps between putative Dal81 or Stp2 target genes identified by ChIP-seq and DEGs in *dal81Δ/Δ* or *stp2Δ/Δ* relative to WT (SN250), as determined by RNA-seq. **(C)** Network view of Dal81 and Stp2 target genes. Genes regulated and bound by Dal81 (left, light cyan), Stp2 (right, mint green), or both (middle, light peach) were presented. Activation was indicated by light coral lines, repression by light blue lines. Targets involved in amino acid metabolism (soft apricot), carboxylic acid metabolism (light coral), organic aid metabolism (light teal), oxoacid metabolism (sage green), and others (dusty pink) were shaded accordingly. Genes further analyzed in Fig. 7E

and F were colored red. The network was visualized using the ggraph and scatterpie R packages. Targets without common names are denoted by unique numerical identifiers (*e.g.*, ‘‘04720’’ for *CAWG*_04720).

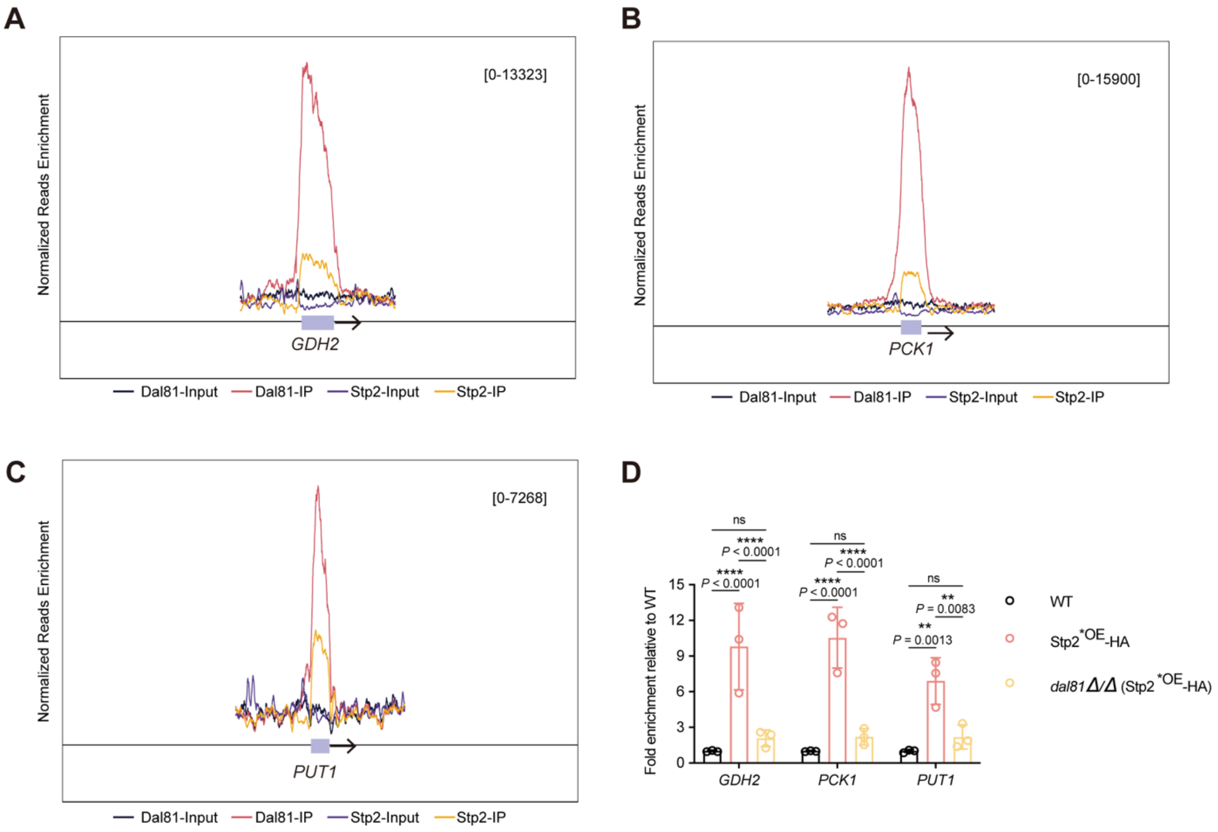

Supplementary Fig. 14 Binding of Dal81 and Stp2 to the promoters of downstream targets (*GDH2*, *PUT1* and *PCK1*). ChIP assays were performed using wild-type *C. albicans* strains expressing epitope-tagged Dal81-Myc or Stp2-HA. Input DNA was included to assess nonspecific binding for anti-Myc and anti-HA antibodies. **(A-C)** ChIP-seq data visualized with MochiView for gene loci of *GDH2* (A), *PUT1* (B), and *PCK1* (C). Red peaks indicate Dal81-Myc enrichment and yellow peaks indicate Stp2-HA enrichment. Black and purple lines represent input DNA for Dal81-Myc and Stp2-HA, respectively. The X-axis denotes a 13.4 kb genomic window; the Y-axis shows enrichment relative to average background signal. Genes are depicted as light purple boxes with black arrows indicating transcriptional orientation. **(D)** ChIP–qPCR validation of Stp2 binding to *GDH2*, *PUT1*, and *PCK1* promoters in Stp2-HA overexpression strains, with or without the *dal81Δ/Δ* background. Binding is expressed as fold enrichment relative to input DNA. The untagged wild-type strain served as a negative control. Data are mean ± SD of three biological replicates. Statistical significance was determined using two-way ANOVA with Tukey's test. ns, not significant. Source data (D) are provided in the Source Data file.

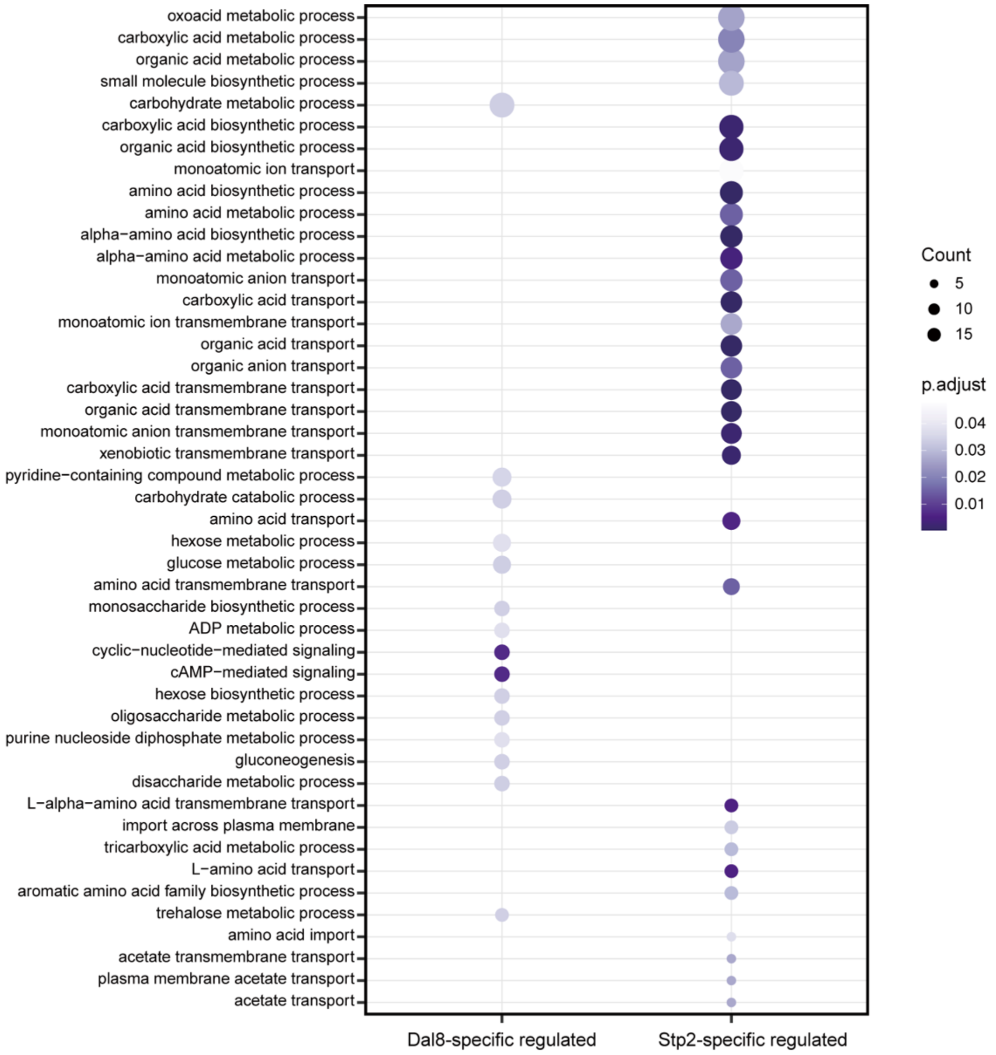

Supplementary Fig. 15 Gene Ontology (GO) enrichment analysis of the alkalization-responsive genes specifically regulated by Dal81 and Stp2.

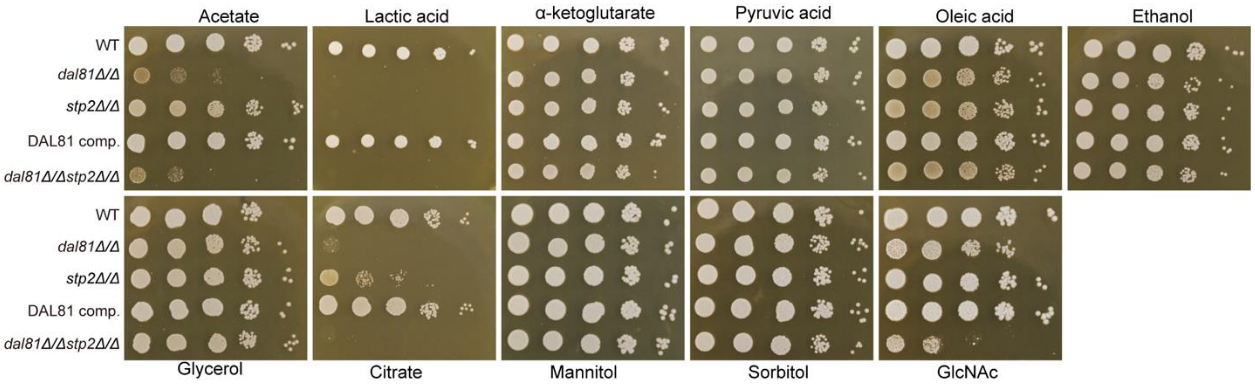

Supplementary Fig. 16 Growth characteristics of *C. albicans* wild-type and the indicated mutant strains on various alternative carbon sources. Overnight cultures were adjusted to an OD_{600} of 1.0, followed by 10-fold serial dilutions. Aliquots (5 µl) of each dilution were spotted onto YEP agar plates supplemented with the indicated alternative carbohydrate sources: 2% acetate, 2% lactic acid, 10 mM α-ketoglutarate, 10 mM pyruvic acid, 0.2% oleic acid, 2% ethanol, 2% glycerol, 2% citrate, 2% mannitol, 2% sorbitol, and 2% N-acetylglucosamine (GlcNAc). Plates were incubated at 37°C for 2 days before photograph.

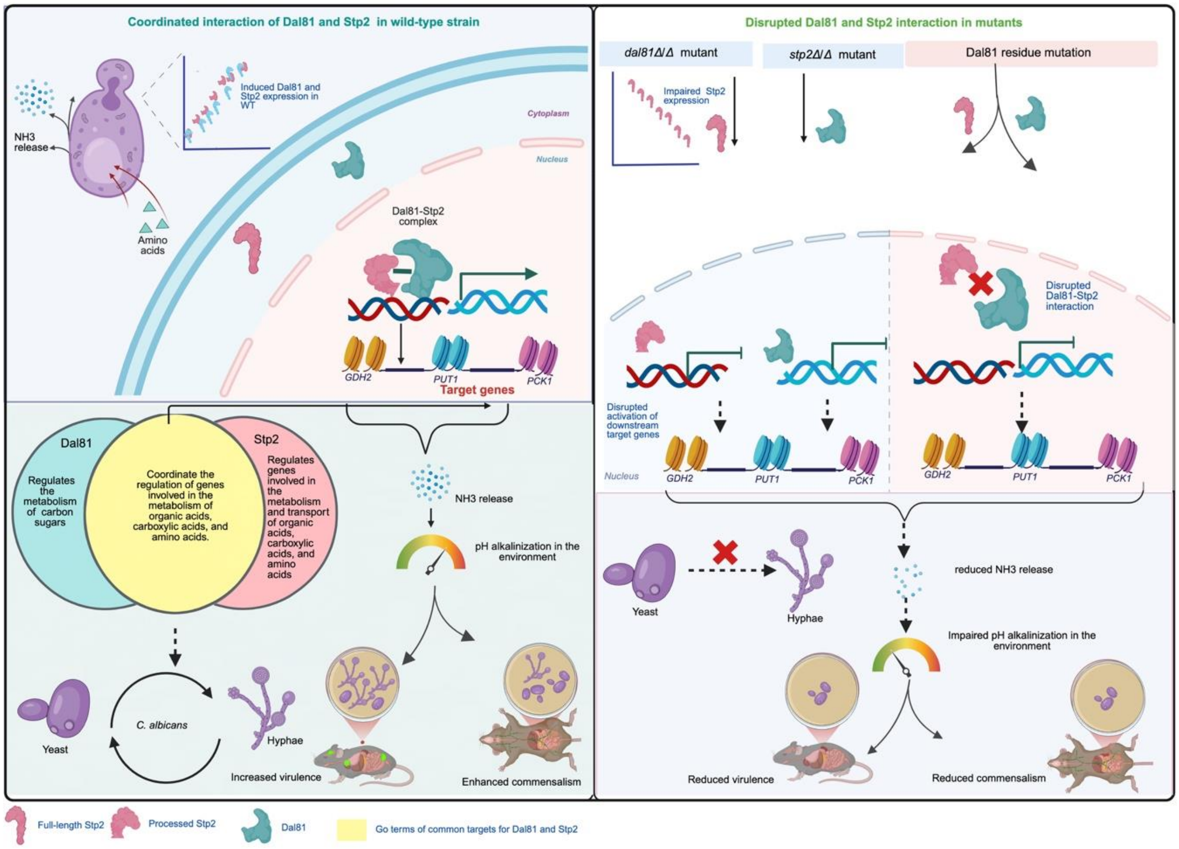

Supplementary Fig. 17 A schematic model illustrating the coordinated regulatory mechanism essential for alkalinization, which plays a pivotal role in modulating *C. albicans* commensalism and pathogenicity.

During amino acid (AA) growth, Dal81 and Stp2 are up-regulated in wild-type strains, localizing to the nucleus and cytoplasm, respectively. Processed Stp2 subsequently translocates into the nucleus, forming a complex with Dal81.This interaction directly activates downstream target genes (*e.g*., *GDH2*), regulating alkalinization via ammonia release. This process is critical for *in vivo* hyphal growth, further promoting commensalism and pathogenicity. In *dal81Δ/Δ* mutants, Stp2 protein levels are significantly reduced but retain processing and nuclear entry capacity. However, processed Stp2 fails to bind downstream genes, blocking extracellular amino acid uptake and halting alkalinization. This prevents *in vivo* hyphal switching, reducing commensalism and pathogenicity. In *stp2Δ/Δ* mutants, Dal81 binding to downstream genes is also impaired, similarly blocking amino acid uptake and alkalinization, leading to defective hyphae switching and attenuated commensalism and pathogenicity traits. The model also highlights an Dal81 mutant with 21 residue mutations required for Dal81-Stp2 interaction and pH alkalinization. These mutations disrupt the interaction, causing alkalinization defects and reduced *C. albicans* virulence. The model was created by BioRender software[1].

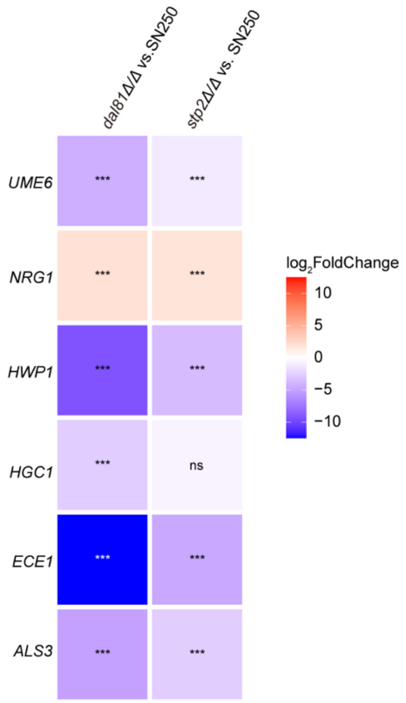

Supplementary Fig. 18 Heatmap displaying expression level changes of selected hyphae-specific genes. *** *p. adjust* < 0.001; ns, not significant.

Supplementary Table 1 Plasmids used in this study

Plasmid	Insert	Purpose	Reference
bCB 60	*pGADT7/AD*	Y2H Gold system empty vector	Clontech
bCB61	*pGADT7/T*	Y2H Gold system postive control	Clontech
bCB62	*pGBKT7/BD*	Y2H Gold system empty vector	Clontech
bCB63	*pGBKT7/p53*	Y2H Gold system postive control	Clontech
bCB137	*PmeI-STP2 C-terminal ORF sequence-3xHA-FRT-FLP-SAT1-FRT- STP2 downstream sequence-PmeI, ligated into pRS316 plasmid*	HA-tagged Stp2	this study
bCB138	*PmeI-DAL81 C-terminal ORF sequence-13xMyc-FRT-FLP-SAT1-FRT- DAL81 downstream sequence-PmeI, ligated into pRS316 plasmid*	Myc-tagged Dal81	this study
bCB155	*XhoI-5' flank of LEU2-DAL81 terminator-ORF-promoter cassette-ARG4 marker-3' flank of LEU2-KpnI*	*DAL81* Addback	this study
bCB 335	*XmaI- Dal81ORF-BamHI, ligated into pGADT7 plasmid*	Yeast 2-Hybrid	this study
bCB336	*BamHI-Stp2ORF-PstI, ligated into pGBKT7 plasmid*	Yeast 2-Hybrid	this study
bCB537	*KpnI -DAL81m21-13xMyc-XhoI, ligated into* pUC-GW/Amp vector	Synthesize the gene fragment of *DAL81m21-13xMyc*	GENEWIZ Gene synthesis
bCB556	*XhoI-5' flank of LEU2-13xMyc-DAL81 ORF-promoter cassette-ARG4 marker-3' flank of LEU2-KpnI*	*DAL81-13xMyc* Addback	this study
bCB557	*XhoI-5' flank of LEU2-13xMyc-DAL81 mutant 21 ORF-promoter cassette-ARG4 marker-3' flank of LEU2-KpnI*	*DAL81m21-13xMyc* Addback	this study
bCB558	*XhoI-5' flank of LEU2-13xMycC- DAL81 mutant 2 ORF-promoter cassette-ARG4 marker-3' flank of LEU2-KpnI*	*DAL81m2-13xMyc* Addback	this study

Reference

1. Hussain, Z. Created in BioRender. https://BioRender.com/8no2un8 (2025).

Images of uncropped Western blots

Fig.3B

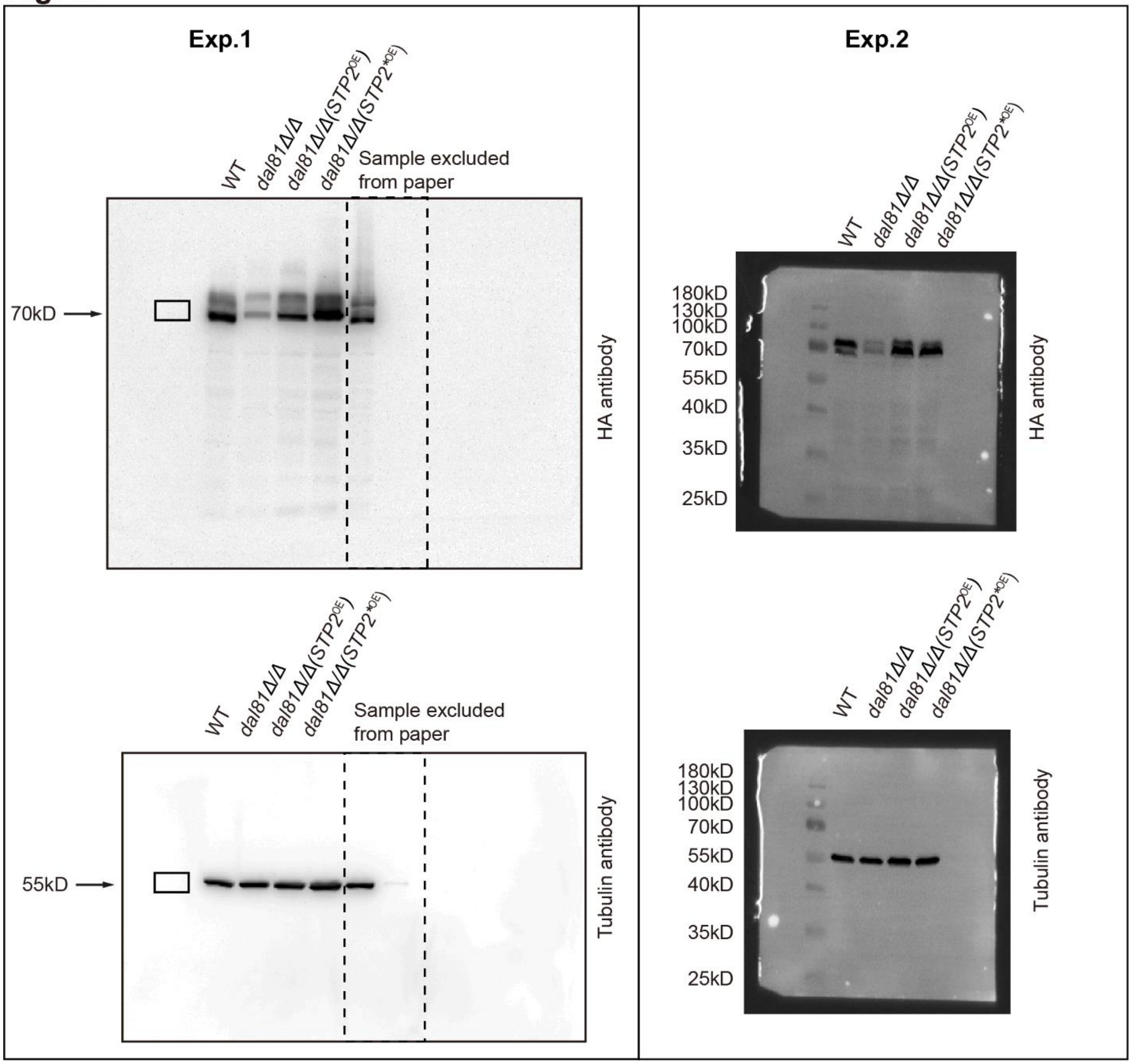

Fig.3D

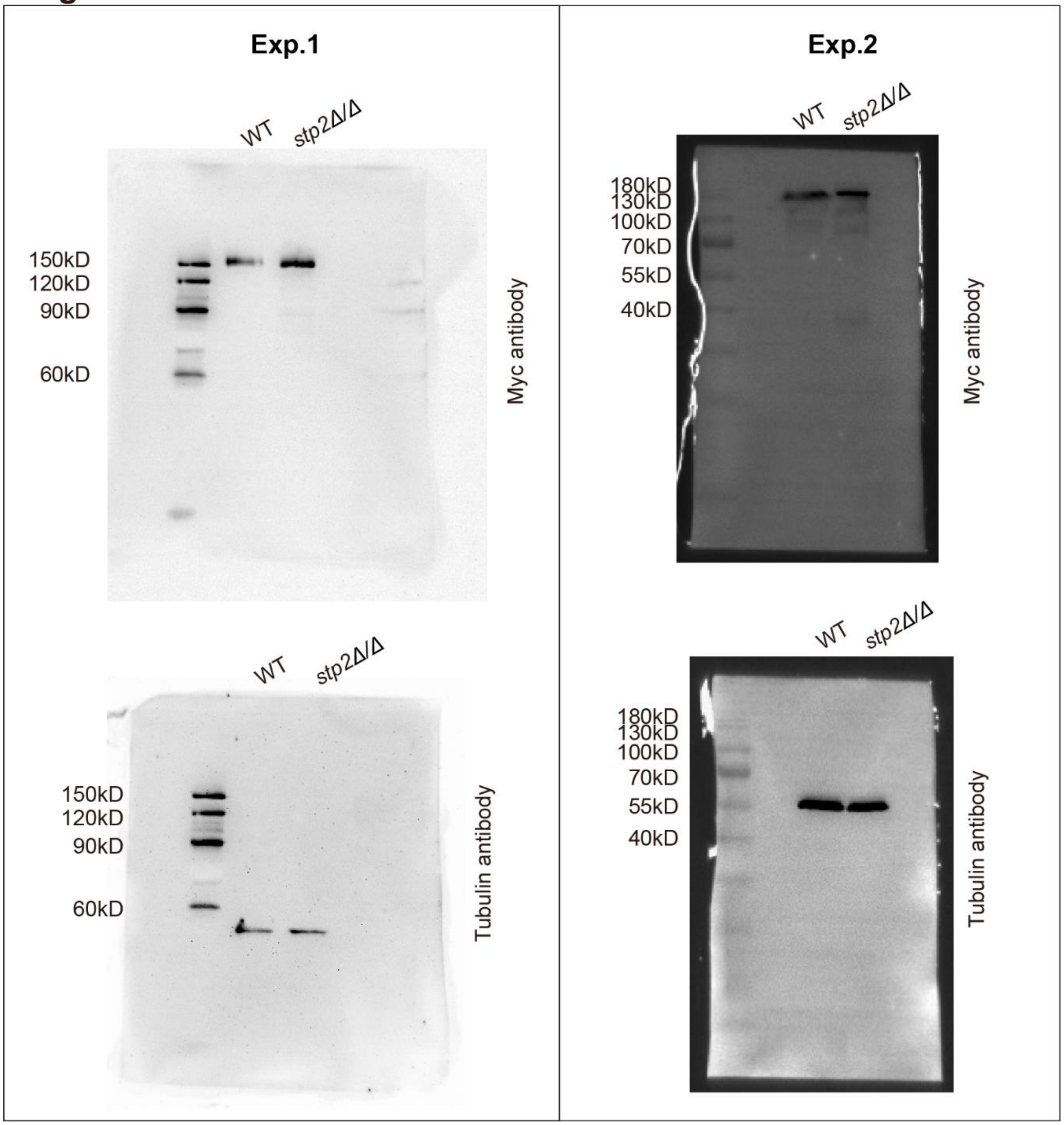

Exp.1
WT
stp2Δ/Δ
150kD
120kD
90kD
60kD
Myc antibody
WT
stp2Δ/Δ
150kD
120kD
90kD
60kD
Tubulin antibody
Exp.2
WT
stp2Δ/Δ
180kD
130kD
100kD
70kD
55kD
40kD
Myc antibody
WT
stp2Δ/Δ
180kD
130kD
100kD
70kD
55kD
40kD
Tubulin antibody

Fig.4C

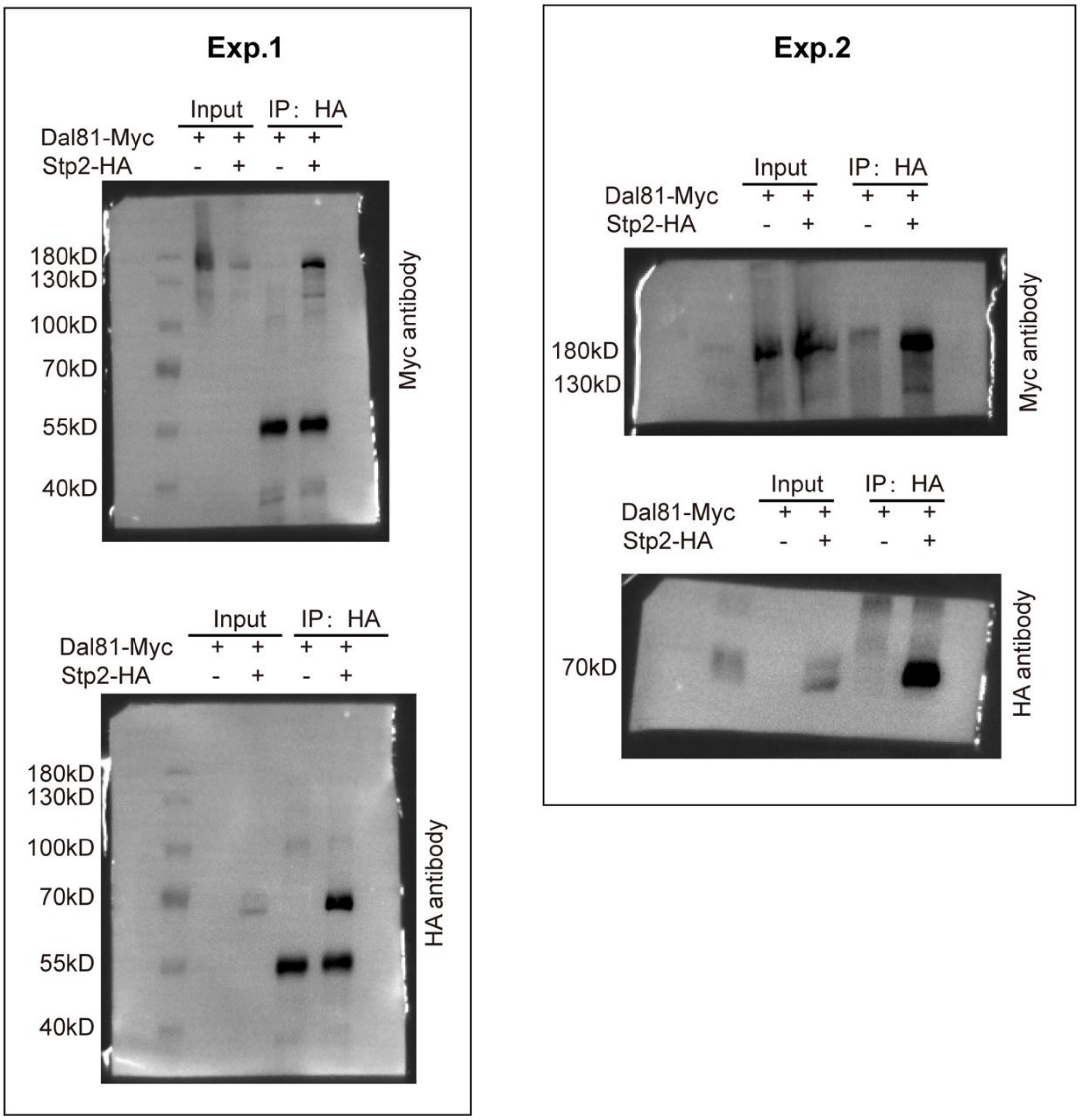
Exp.1
Input
IP：HA
Dal81-Myc
Stp2-HA
180kD
130kD
100kD
70kD
55kD
40kD
Myc antibody
Input
IP：HA
Dal81-Myc
Stp2-HA
180kD
130kD
100kD
70kD
55kD
40kD
HA antibody
Exp.2
Input
IP：HA
Dal81-Myc
Stp2-HA
180kD
130kD
Myc antibody
Input
IP：HA
Dal81-Myc
Stp2-HA
70kD
HA antibody

Fig.4D

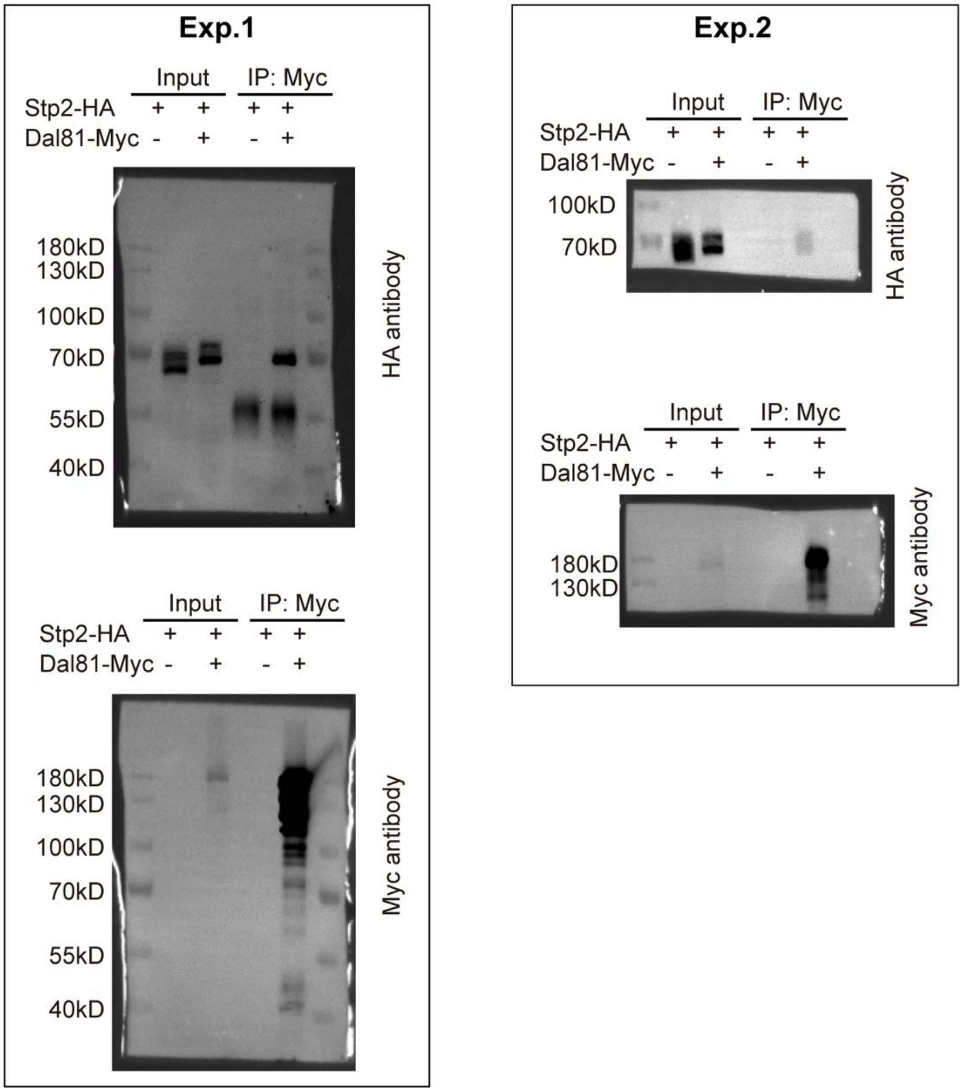
Exp.1
Input
IP: Myc
Stp2-HA + + + +
Dal81-Myc - + - +
180kD
130kD
100kD
70kD
55kD
40kD
HA antibody
Input
IP: Myc
Stp2-HA + + + +
Dal81-Myc - + - +
180kD
130kD
100kD
70kD
55kD
40kD
Myc antibody
Exp.2
Input
IP: Myc
Stp2-HA + + + +
Dal81-Myc - + - +
100kD
70kD
HA antibody
Input
IP: Myc
Stp2-HA + + + +
Dal81-Myc - + - +
180kD
130kD
Myc antibody

Fig.5C

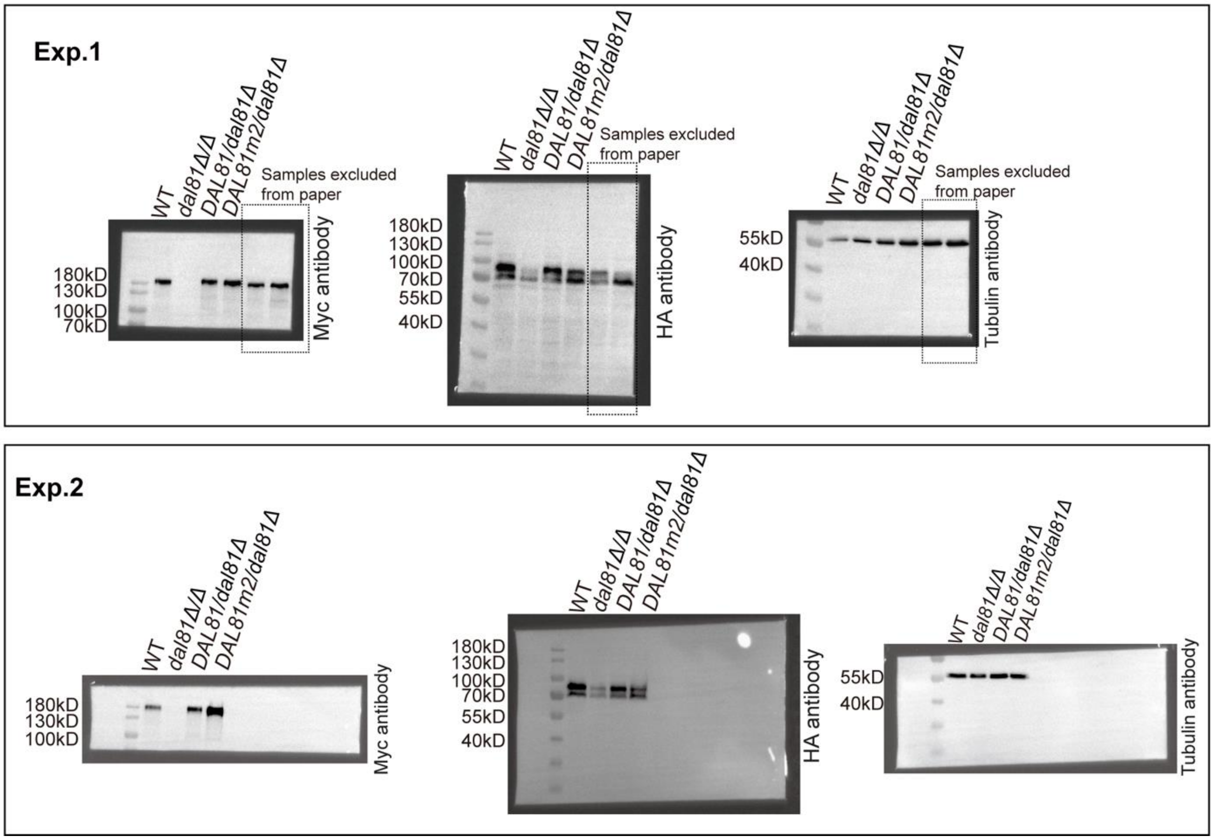

Exp.1
WT
dal81Δ/Δ
DAL81/dal81Δ
DAL81m2/dal81Δ
Samples excluded from paper
180kD
130kD
100kD
70kD
Myc antibody
55kD
40kD
HA antibody
Tubulin antibody
Exp.2

Fig.5G

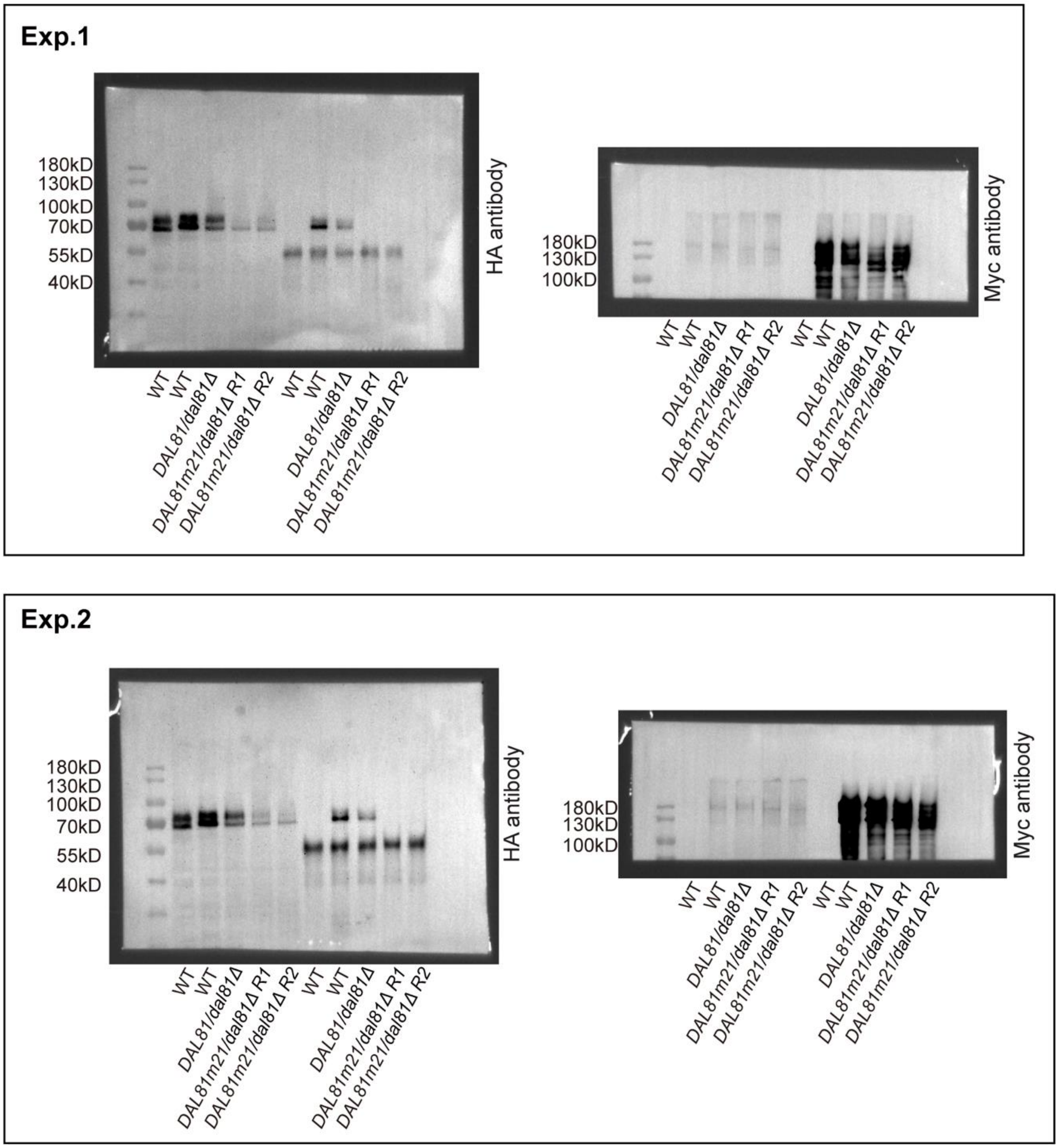

Exp.1
180kD
130kD
100kD
70kD
55kD
40kD
HA antibody
WT
WT
DAL81/dal81Δ
DAL81m21/dal81Δ R1
DAL81m21/dal81Δ R2
WT
WT
DAL81/dal81Δ
DAL81m21/dal81Δ R1
DAL81m21/dal81Δ R2
180kD
130kD
100kD
Myc antibody
WT
WT
DAL81/dal81Δ
DAL81m21/dal81Δ R1
DAL81m21/dal81Δ R2
WT
WT
DAL81/dal81Δ
DAL81m21/dal81Δ R1
DAL81m21/dal81Δ R2
Exp.2
180kD
130kD
100kD
70kD
55kD
40kD
HA antibody
WT
WT
DAL81/dal81Δ
DAL81m21/dal81Δ R1
DAL81m21/dal81Δ R2
WT
WT
DAL81/dal81Δ
DAL81m21/dal81Δ R1
DAL81m21/dal81Δ R2
180kD
130kD
100kD
Myc antibody
WT
WT
DAL81/dal81Δ
DAL81m21/dal81Δ R1
DAL81m21/dal81Δ R2
WT
WT
DAL81/dal81Δ
DAL81m21/dal81Δ R1
DAL81m21/dal81Δ R2
