# Supplementary material for: Coordinated regulation of pH alkalinization by two transcription factors promotes fungal commensalism and pathogenicity
Source: Nat Commun. 2025 Aug 22;16:7855. doi: 10.1038/s41467-025-62953-x (PMC12373999; doi:10.1038/s41467-025-62953-x)
Supplement: Supplementary file 2 — Description of Additional Supplementary Files [file 41467_2025_62953_MOESM2_ESM.pdf]

Title: Supplementary Data 1

Description: RNA-seq analysis reveals pH Alkalinization -responsive genes in *C. albicans*. This table shows the differentially expressed genes (DEG), log2 fold change (log2FC≥1), and FDR adjusted p value (padj <0.05, DESeq2) in SC5314 cultured in SD medium for 6 hours versus SC5314 cultured in YNB + 1% CAA medium (with an initial pH of 4.5) for 6 hours. Alkalinization-responsive gene sets controlled by Dal81 and Stp2 based on RNA-seq analysis of the respective knockout mutants versus SN250. Gene IDs and gene names are derived from Candida Genome Database Assembly 22.

Title: Supplementary Data 2

Description: GO terms of alkalinization-responsive genes. This table shows the GO enrichment results for Biological Processes of all the alkalinization-responsive genes, as well as those that are Dal81-dependent, Stp2-dependent, and co-dependent on Dal81 and Stp2.

Title: Supplementary Data 3

Description: Dal81 and Stp2 targets identified by ChIP-seq.

Title: Supplementary Data 4

Description: Target Genes of Dal81 and Stp2. This table shows Dal81-Myc and Stp2-HA bound and expression level altered genes.

Title: Supplementary Data 5

Description: GO terms of common targets for Dal81 and Stp2. This table shows the GO enrichment results for Biological Processes of common targets of Dal81 and Stp2

Title: Supplementary Data 6

Description: GO terms of specific genes for Dal81 and Stp2. This table shows the GO enrichment results for Biological Processes of genes specifically regulated by Dal81 or Stp2.

Title: Supplementary Data 7

Description: Strains used in this study.

Title: Supplementary Data 8

Description: Primers used in this study.
