# Supplementary material for: Coordinated regulation of pH alkalinization by two transcription factors promotes fungal commensalism and pathogenicity
Source: Nat Commun. 2025 Aug 22;16:7855. doi: 10.1038/s41467-025-62953-x (PMC12373999; doi:10.1038/s41467-025-62953-x)
Supplement: Supplementary file 11 — Reporting Summary [file 41467_2025_62953_MOESM11_ESM.pdf]

nature portfolio

Corresponding author(s): Xinhua Huang, Hao Li, Changbin Chen

Last updated by author(s): Aug 1, 2025

Reporting Summary

Nature Portfolio wishes to improve the reproducibility of the work that we publish. This form provides structure for consistency and transparency in reporting. For further information on Nature Portfolio policies, see our Editorial Policies and the Editorial Policy Checklist.

Statistics

For all statistical analyses, confirm that the following items are present in the figure legend, table legend, main text, or Methods section.

n/a	Confirmed	
[ ]	[x]	The exact sample size (n) for each experimental group/condition, given as a discrete number and unit of measurement
[ ]	[x]	A statement on whether measurements were taken from distinct samples or whether the same sample was measured repeatedly
[ ]	[x]	The statistical test(s) used AND whether they are one- or two-sided<br>*Only common tests should be described solely by name; describe more complex techniques in the Methods section.*
[x]	[ ]	A description of all covariates tested
[ ]	[x]	A description of any assumptions or corrections, such as tests of normality and adjustment for multiple comparisons
[ ]	[x]	A full description of the statistical parameters including central tendency (e.g. means) or other basic estimates (e.g. regression coefficient) AND variation (e.g. standard deviation) or associated estimates of uncertainty (e.g. confidence intervals)
[ ]	[x]	For null hypothesis testing, the test statistic (e.g. F, t, r) with confidence intervals, effect sizes, degrees of freedom and P value noted<br>*Give P values as exact values whenever suitable.*
[x]	[ ]	For Bayesian analysis, information on the choice of priors and Markov chain Monte Carlo settings
[x]	[ ]	For hierarchical and complex designs, identification of the appropriate level for tests and full reporting of outcomes
[x]	[ ]	Estimates of effect sizes (e.g. Cohen's d, Pearson's r), indicating how they were calculated

Our web collection on statistics for biologists contains articles on many of the points above.

Software and code

Policy information about availability of computer code

Data collection	FV10-ASW v04.02.03.06, Olympus cellSens Dimension 2.3
Data analysis	GraphPad Prism 10.0, Imaris 9.5.1, ImageJ Fiji v2.3, Adobe PhotoShop 2022, FastQC, MultiQC v1.13, Trimmomatic v0.39, STAR 2.7.6a, featureCounts v2.0.1, Bowtie2 v1.3.1, Deeptools 3.5.1, Sambamba v0.7.1, Samtools v1.6, Macs2 v2.2.7.1, ChimeraX, R v4.3, R package DESeq2 v1.20, R package ComplexHeatmap v2.20.0, R package clusterProfiler.

For manuscripts utilizing custom algorithms or software that are central to the research but not yet described in published literature, software must be made available to editors and reviewers. We strongly encourage code deposition in a community repository (e.g. GitHub). See the Nature Portfolio guidelines for submitting code & software for further information.

Data

Policy information about availability of data

All manuscripts must include a data availability statement. This statement should provide the following information, where applicable:

- Accession codes, unique identifiers, or web links for publicly available datasets
- A description of any restrictions on data availability
- For clinical datasets or third party data, please ensure that the statement adheres to our policy

Dal81 ChIP-Seq, Stp2 ChIP-Seq and RNA-Seq data have been deposited in the National Center for Biotechnology Information Sequence Read Archive with accession number PRJNA1190138 (https://www.ncbi.nlm.nih.gov/bioproject/PRJNA1190138/). Source data are provided with this paper.

Research involving human participants, their data, or biological material

Policy information about studies with human participants or human data. See also policy information about sex, gender (identity/presentation), and sexual orientation and race, ethnicity and racism.

Reporting on sex and gender	n/a
Reporting on race, ethnicity, or other socially relevant groupings	n/a
Population characteristics	n/a
Recruitment	n/a
Ethics oversight	n/a

Note that full information on the approval of the study protocol must also be provided in the manuscript.

Field-specific reporting

Please select the one below that is the best fit for your research. If you are not sure, read the appropriate sections before making your selection.

[x] Life sciences [ ] Behavioural & social sciences [ ] Ecological, evolutionary & environmental sciences

For a reference copy of the document with all sections, see nature.com/documents/nr-reporting-summary-flat.pdf

Life sciences study design

All studies must disclose on these points even when the disclosure is negative.

Sample size	No statistical methods were used to predetermine sample size. Sample sizes were estimated based on preliminary experiments and field standards. For in vitro experiments, 3 samples were analyzed; For the ChIP-seq assay, two biological immunoprecipitation (IP) samples were used for sequencing. For mouse experiments, 8-12 mice per group were used. These sample sizes were sufficient to detect meaningful biological differences with acceptable reproducibility.
Data exclusions	No data were excluded.
Replication	All results were repeated at least twice with similar result, except for the screen (n=1).
Randomization	Samples/ mice were randomly allocated to different experimental groups and included in the experiments.
Blinding	Investigators were not blinded to group allocation during data collection and analysis, as the perceived risk of performance bias was considered negligible. However, RNA-seq and ChIP-seq analyses were conducted in a blinded manner using the software tools.

Reporting for specific materials, systems and methods

We require information from authors about some types of materials, experimental systems and methods used in many studies. Here, indicate whether each material, system or method listed is relevant to your study. If you are not sure if a list item applies to your research, read the appropriate section before selecting a response.

Materials & experimental systems

n/a	Involved in the study	
[ ]	[x]	Antibodies
[ ]	[x]	Eukaryotic cell lines
[x]	[ ]	Palaeontology and archaeology
[ ]	[x]	Animals and other organisms
[x]	[ ]	Clinical data
[x]	[ ]	Dual use research of concern
[x]	[ ]	Plants

Methods

n/a	Involved in the study	
[ ]	[x]	ChIP-seq
[x]	[ ]	Flow cytometry
[x]	[ ]	MRI-based neuroimaging

Antibodies

Antibodies used	Anti-Myc antibody (MBL, Cat# M192-3) – Used at 1:10,000 dilution for Western blot (WB).<br><br>Anti-Myc (19C2) antibody (Abmart, Cat# M20002) – Used at 1:200 dilution per IP sample for ChIP assay.

Anti-HA (3F10) antibody (Roche Diagnostics, Cat# 11867423001) – Used at 4 µg/ml per IP sample for ChIP assay and 100 ng/ml for WB.

Anti-tubulin antibody (Novus Biologicals, Cat# NB100-1639) – Used at 1:2,000 dilution for WB.

EZview™ Red anti-c-Myc affinity gel (Sigma, Cat# E6654) – Used at 50 µl per IP sample for Co-IP assay.

EZview™ Red anti-HA affinity gel (Sigma, Cat# E6779) – Used at 50 µl per IP sample for Co-IP assay.

Validation

https://www.mblbio.com/bio/g/dtl/A/index.html?pcd=M192-3
chrome-extension://efaidnbmnnnibpcajpcglclefindmkaj/https://www.ab-mart.com.cn/upload/20170614093526xz.pdf
https://www.sigmaaldrich.com/TW/en/search/roche-11867423001?
focus=products&page=1&perpage=30&sort=relevance&term=roche%2011867423001&type=product
https://www.novusbio.com/support/protocols/western-blot-protocol-for-alpha-tubulin-antibody-nb100-1639.html?
srsltid=AfmBOoopZlUZPutBsmhbH1IKt7TmEnR6RpTe8ynaqKzMRQLZkNBDVZP7
https://www.sigmaaldrich.com/TW/en/product/sigma/e6654?
srsltid=AfmBOopqjWes3oumKIItQtYE2MRiAPz9aFTR5wQ8rBq10T70UFgQFaU_
https://www.sigmaaldrich.com/TW/en/product/sigma/e6779?
srsltid=AfmBOorVfOXfoWjuvIntDBprc44BL60FWSqpvgia3gTy4avc0OiIs1RS

Eukaryotic cell lines

Policy information about cell lines and Sex and Gender in Research

Cell line source(s)

RAW264.7 cell line(ATCC TIB-71), J774A.1 cell line (ATCC TIB-67). BMDM cells used were isolated in this study.

Authentication

All cell line used were not authorized.

Mycoplasma contamination

Cell lines were not tested for mycoplasma contamination; however, no signs of contamination were observed during the course of the experiments.

Commonly misidentified lines
(See ICLAC register)

Nocommonly misidentified cell lines are used.

Animals and other research organisms

Policy information about studies involving animals; ARRIVE guidelines recommended for reporting animal research, and Sex and Gender in Research

Laboratory animals

Female C57BL/6 mice (6–8 weeks old, 18–20 g) and female ICR mice (6–8 weeks old, 23–25g) were purchased from Beijing Vital River Laboratory Animal Technology Company (Beijing, China). All mice were housed in a pathogen-free animal facility under controlled conditions (temperature: 21°C; relative humidity: 50–70%; 12-hour light/dark cycle) and had ad libitum access to food and water. Mice were given free access to food and water throughout the study. All experimental procedures were conducted in accordance with the protocol approved by the Institutional Animal Care and Use Committee (IACUC) at Institut Pasteur of Shanghai, Chinese Academy of Sciences, China.

Wild animals

The study did not involve wild animals.

Reporting on sex

The study did not involve sex-based analysis.

Field-collected samples

The study did not involve samples collected from the field.

Ethics oversight

The animal experiments performed in this study were approved by Institutional Animal Care and Use Committee (IACUC) at the Shanghai Institute of Immunity and Infection, Chinese Academy of Sciences (Permit Number: A2020025).

Note that full information on the approval of the study protocol must also be provided in the manuscript.

Plants

Seed stocks

n/a

Novel plant genotypes

n/a

Authentication

n/a

ChIP-seq

Data deposition

- [x] Confirm that both raw and final processed data have been deposited in a public database such as GEO.
- [ ] Confirm that you have deposited or provided access to graph files (e.g. BED files) for the called peaks.

Data access links<br>*May remain private before publication.*	Dal81 ChIP-Seq and Stp2 ChIP-Seq data have been deposited in the National Center for Biotechnology Information Sequence Read Archive with accession number PRJNA1190138 (https://www.ncbi.nlm.nih.gov/bioproject/PRJNA1190138/).
Files in database submission	Files "H1" and "H2" represent two biological replicates of immunoprecipitated chromatin from Dal81-13xMyc strains. File "H3" denotes the total chromatin from SN250, serving as the untagged control. Files "H4" and "H5" are two biological replicates of immunoprecipitated chromatin from Stp2-3xHA strains, while file "H6" represents the total chromatin from SN250 as the untagged control.
Genome browser session<br>(e.g. UCSC)	The reference genome we used is C. albicans SC5314 (Assembly 22) genome , available on the Candida genome database.

Methodology

Replicates	There are two biological replicates of immunoprecipitated chromatin from Dal81-13xMyc strains and two biological replicates from Stp2-3xHA strains.
Sequencing depth	1.4 x10^8/sample
Antibodies	Mouse anti-Myc (19C2) antibody (Abmart, M20002), Rat anti-HA(3F10) antibody(Roche Diagnostics, 11867423001).
Peak calling parameters	-g 1.3e+7
Data quality	Data quality was ensured through the use of fastqc to ensure read quality.
Software	Trimmomatic v0.39, FastQC, BBowtie2 v1.3.1, Sambamba v0.7.1, Samtools v1.6, Macs2 v2.2.7.1.
